# *EGFR*, *PIK3CA*, *KRAS* and *BRAF* mutations in meningiomas

**DOI:** 10.3892/ol.2014.2042

**Published:** 2014-04-07

**Authors:** MATEUSZ BUJKO, PAULINA KOBER, ANDRZEJ TYSAROWSKI, EWA MATYJA, TOMASZ MANDAT, WIESŁAW BONICKI, JANUSZ ALEKSANDER SIEDLECKI

**Affiliations:** 1 Department of Molecular and Translational Oncology, Maria Sklodowska-Curie Memorial Cancer Center and Institute of Oncology, Warsaw 02-781, Poland; 2 Department of Experimental and Clinical Neuropathology, M. Mossakowski Medical Research Centre, Polish Academy of Sciences, Warsaw 02-106, Poland; 3 Department of Neurosurgery, Maria Sklodowska-Curie Memorial Cancer Center and Institute of Oncology, Warsaw 02-781, Poland

**Keywords:** meningioma, *EGFR*, *PI3K*, *PIK3CA*, mutations

## Abstract

Meningiomas are among the most frequent intracranial tumors. Treatment involves surgical resection with optional subsequent radiotherapy for high-grade meningiomas or radiosurgery following incomplete tumor removal. At present, no pharmacological agents are used as treatment. The use of targeted therapies has been considered, and specific therapies, including anti-EGFR treatment, have been clinically tested. The experience from the treatment of various types of cancers shows that patient outcome depends on the mutational status of particular molecules, including epithelial growth factor receptor (EGFR), Kirsten rat sarcoma viral oncogene homolog (KRAS), v-Raf murine sarcoma viral oncogene homolog B1 (BRAF) and phosphatidylinositol-4,5-bisphosphate 3-kinase, catalytic subunit α (PIK3CA). Therefore, the aim of the present study was to assess the occurrence and potential use of these markers in patients with meningioma. In total, 55 formalin-fixed, paraffin-embedded meningioma samples were subjected to genomic sequencing of *EGFR* (exons 18–21), *KRAS* (exon 1), *BRAF* (exon 15) and PI3K (exons 9, 20). No mutations were identified in *EGFR*, *KRAS* or *BRAF*. Point mutations in PIK3CA were revealed in the samples of two patients with atypical and anaplastic meningiomas. Although these mutations appear to be rare, this result, along with previously reported findings, indicates that the PI3K/protein kinase B pathway may serve as a more reasonable molecular target for meningioma than EGFR.

## Introduction

Meningiomas are one of the most frequent intracranial tumors. The majority of meningiomas are benign lesions of World Health Organization (WHO) grade I (GI), however, certain histopathological variants are associated with more aggressive clinical behavior and correspond to WHO GII and III (1). The GII and III meningiomas have an increased risk of local recurrence. The standard treatment for high-grade meningiomas involves surgical resection with optional radiotherapy, and radiosurgery in the case of an incomplete resection. No pharmacological agents are routinely used for the treatment of meningiomas. The epithelial growth factor receptor (EGFR) is one of the most extensively studied molecular targets for anti-cancer therapy and is also considered as a target for meningioma treatment (2). This receptor is expressed in most meningioma patients and was initially identified to be involved in tumor development (3). The results of studies on *EGFR* expression in meningiomas are not entirely consistent. The percentage of EGFR-immunopositive tumors range between 50% (n=85) (4) and 89% (n=132) (5). In addition to the expression status, receptor activation has also been shown in patients (6,7). In the IOMM-Lee meningioma cell line, the EGFR pathway was shown to be involved in radiation-induced progression (8). EGFR activates several downstream pathways, primarily those of mitogen-activated protein kinase (MAPK), phosphatidylinositol-4,5-bisphosphate 3-kinase/protein kinase B (PI3K/AKT) and phospholipase C, which have been found to play a role in meningioma pathogenesis (7,9).

Based on this background, two of the low molecular weight anti-EGFR inhibitors, gefitinib and erlotinib, were introduced in phase II clinical trials in patients with recurrent meningioma (North American Brain Tumor Consortium Trials 00–01 and 01–03). In these studies, small groups of patients were included (n=9 and n=16, respectively) and no clear effect of the treatment was observed (10).

The data from previous clinical trials on the treatment of other tumor types showed that clinical response may depend on the presence of the mutations in *EGFR* (11) and in genes encoding downstream proteins involved in the signal transduction, including Kirsten rat sarcoma viral oncogene homolog (*KRAS*), v-Raf murine sarcoma viral oncogene homolog B1 (*BRAF*) or phosphatidylinositol-4,5-bisphosphate 3-kinase, catalytic subunit α (*PIK3CA*) (12). Mutations of the *EGFR* tyrosine kinase (TK) domains lead to constitutive kinase activation and were shown to be a good predictive marker in non-small cell lung cancer, while *KRAS* and *BRAF* were negative prognostic markers in colorectal cancer (12,13). *PIK3CA* encodes the catalytic subunit of the PI3K protein and is affected by gain of function mutations in a significant ratio of solid tumors (14). The role of *PIK3CA* mutations in anti-EGFR therapy has not been definitively clarified. In colorectal cancer they coexist with *KRAS* mutations and are considered to be co-responsible for the resistance to targeted therapy (12). The mutations in the aforementioned downstream proteins generally cause activation of the MAPK or AKT pathways independent of the ligand binding or receptor status, and in lung cancer they have been shown to be mutually exclusive with *EGFR* mutations (15).

Previous meningioma studies have shown that *EGFR* does not undergo gene amplification (16,17), but to the best of our knowledge the point mutations in the TK domain of the receptor, which is encoded by exons 18–21, have not been analyzed. Each of the *BRAF*, *KRAS* and *PI3K* genes has been analyzed once previously (18–20). The present study aimed to assess the occurrence of the mutations in the *EGFR* TK domain and in the selected downstream genes, *KRAS*, *BRAF* and *PIK3CA*, in a relatively large group of meningioma patients.

## Materials and methods

### Patients and tissue samples

Tissue samples used for DNA isolation and genomic sequencing consisted of archival formalin-fixed, paraffin-embedded (FFPE) meningioma specimens from patients who underwent surgical tumor resection at the Department of Neurosurgery, M. Sklodowska-Curie Memorial Oncology Center (Warsaw, Poland) between 2007 and 2012. In total, 55 meningioma samples, including 20 GI, 22 GII and 13 GIII, were used in the study. All tissue samples underwent histopathological examination that involved a review of the initial diagnosis and grading according to the 2007 WHO guidelines (1). The representative tissue samples were selected for molecular analysis. Patient characteristics are listed in Table I. Written informed consent was obtained from the patients and the study was approved by the local ethics committee of M. Sklodowska-Curie Memorial Cancer Center and Institute of Oncology (Warsaw, Poland).

### Identification of genomic mutations

FFPE tissue samples were manually macrodissected and DNA was isolated with the use of a QIAamp DNA mini kit (Qiagen, Hilden, Germany). The purity and concentration of the DNA samples were assessed with the use of NanoDrop 1000 spectrophotometer (Thermo Scientific, Rockford, IL, USA) and with PicoGreen dsDNA quantitation assay (Invitrogen Life Technologies, Carlsbad, CA, USA).

DNA was amplified by polymerase chain reaction (PCR) containing 1× PCR buffer, 2 mM MgCl_2_, 250 μM of each oligonucleotide, 0.15 μM of each PCR primer and 0.5 units of FastStart Taq DNA Polymerase (Roche Diagnostics, Mannheim, Germany). The GeneAmp 9700 PCR system (Applied Biosystems, Foster City, CA, USA) was used with the following cycling conditions: Initial denaturation at 94°C for 3 min, followed by 40 cycles of 30 sec at 94°C, 30 sec at 57ºC and 30 sec at 72°C, and a final elongation for 7 min at 72°C. The PCR products were analyzed by electrophoresis in a 2% agarose gel, visualized with the use of ethidium bromide and subjected to DNA sequencing. Briefly, 2.5 μl of the PCR product was purified with the use of ExoStar (GE Healthcare Life Sciences, Pittsburgh, PA, USA) and labeled with BigDye Terminator v.3.1 (Applied Biosytems) according to the manufacturer’s instructions. The ABI PRISM 3300 Genetic Analyzer (Applied Biosystems) was used for capillary electrophoresis and the sequence readout.

## Results

**Figure 1:**
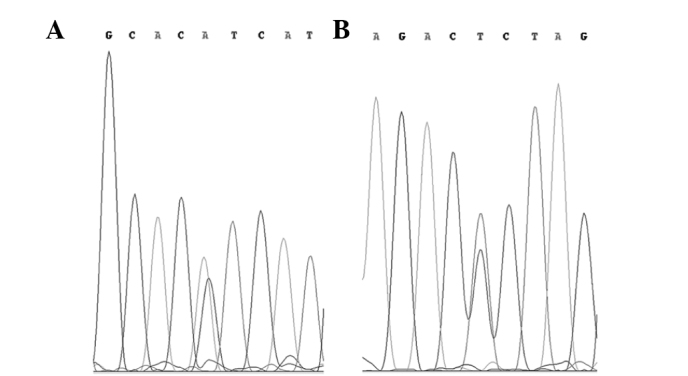
Genomic sequencing electropherogram of the samples with *PIK3CA* mutations. (A) GII meningioma sample with an A>AG, 1047H>H/R mutation in codon 3140. (B) GIII meningioma sample with a C>CT, 1025T>T silent mutation in codon 3075. *PIK3CA*, phosphatidylinositol-4,5-bisphosphate 3-kinase, catalytic subunit α; GII, grade II.

All the DNA regions involved in the sequence analysis (*EGFR* exons 18–21, *KRAS* exon 1, *BRAF* exon 15 and *PI3K* exons 9 and 20) were successfully amplified and sequenced for all the patients enrolled. No mutations were identified in either *EGFR*, *KRAS* or *BRAF*. There were two *PIK3CA* exon 20 mutations identified. These were the missense mutation A>AG, 1047H>H/R in codon 3140, and the silent mutation C>CT, 1025T>T in codon 3075 (Fig. 1). These samples were resected from a 54-year-old male patient with a GII atypical meningioma in the cerebellopontine angle and from a 53-year-old female patient with a GIII anaplastic meningioma located in the cerebellopontine angle, respectively.

## Discussion

The results of the present study are consistent with previously reported studies that also did not identify an incidence of mutations in *KRAS* (17) or *BRAF* (18). The lack of mutations in the *EGFR* TK domain and the downstream proteins that have been observed may indicate a low importance of this pathway in the growth of meningiomas, despite a previously suggested hypothesis (6). This is in line with previous studies that report no incidence of *EGFR* gene amplification in meningiomas (16), as these molecular aberrations have a similar effect on signal transduction. This is also consistent with a previous study reporting that EGFR expression occurs less frequently in malignant meningiomas than in GI patients (5), and EGFR-positive patients have an improved survival prognosis compared with EGFR-negative patients (4).

The purpose of the mutation screening in the present study was to assess whether it is possible to use the molecular background to identify meningioma patients who may have a potentially improved response to anti-EGFR treatment, as is possible in the case of other tumors. The results show that known predictive markers have no value in meningiomas, and the lack of *EGFR* and downstream protein mutations may partially explain the results of the clinical trials with the anti-EGFR inhibitors, erlotinib and gefitinib (10).

All the aforementioned data indicate that despite the fact that EGFR is expressed in meningeal tumors, it does not contribute to tumor development or progression, as was hypothesized initially (9). This in turn indicates that the EGFR pathway is not a promising target for the treatment of meningiomas.

In the present study, the only identified genetic aberrations were the mutations of the kinase domain of *PIK3CA* that were observed in two patients with GII and GIII tumors. The genetic changes, 1047H>H/R and 1025T>T, have already been reported in the COSMIC (Catalog of Somatic Mutations in Cancer) database (database ID 775 and 21451, respectively). The 1047H>H/R missense mutation is the most common mutation reported in tumor samples and accounts for 24.4% of all variations of the *PIK3CA* coding sequence submitted to the COSMIC database. This mutation has revealed strong oncogenic potential *in vivo* (20) and recently was also shown to be associated with the response to PI3K/AKT pathway inhibitors in clinical trials (21). The second identified genomic variation does not affect the amino acid sequence and is less frequent in cancer patients, with a frequency of 0.3% *PIK3CA* mutations reported in the COSMIC database.

The occurrence of the *PIK3CA* mutations in meningiomas was investigated in a previous study, and one of the patients with anaplastic meningioma was identified as a mutation carrier (19). The combination of data from the previous and current studies indicates a 3.4% (3/87) frequency of *PIK3CA* variations in atypical and anaplastic meningiomas. This can be described as rare, but the PI3K/AKT pathway may also be deregulated by the mutations in phosphatase and tensin homolog (*PTEN*); a PI3K inhibitor. In meningiomas, these gene mutations have previously been shown to occur with a frequency similar to the *PIK3CA* mutations (22). In total, two of the *PTEN* mutations were identified in GIII tumors and one in a radiation-induced meningioma. No *PIK3CA* or *PTEN* mutations were found in any GI patients (19,22). This indicates the involvement of this pathway in tumor progression. The phosphorylation in the PI3K/AKT pathway was previously observed as being more frequent in GII and III meningiomas, and pharmacological inhibition of this pathway in primary malignant meningioma cells resulted in reduced proliferation and survival (7).

In the light of all the presented facts it appears that PI3K and its co-regulators may be more reasonable molecular targets in meningiomas than EGFR. Recently, a number of small molecular inhibitors that target PI3K have been reported in clinical trials (21), and mutations of *PIK3CA/PTEN* have been show to have a predictive value in the administration of these compounds (23,24). We believe that these results should encourage clinicians to consider the possible use of this pharmacological treatment in malignant meningiomas.

## Figures and Tables

**Table I:** Patient characteristics.

Characteristics	Value
Patients, n	55
Male	21
Female	34
Age, years	
Range	21–80
Median	55
Grade, n	
GI	30
Meningioma menigotheliale	7
Meningioma fibroblasticum	4
Meningioma transitionale	13
Other	6
GII meningioma atypicum	16
GIII meningioma anaplasticum	9

GI, grade I.
